# Breast cancer subtype discordance: impact on post-recurrence survival and potential treatment options

**DOI:** 10.1186/s12885-018-4101-7

**Published:** 2018-02-20

**Authors:** Peter F. McAnena, James AL Brown, A. Ramli, C. Curran, C. Malone, R. McLaughlin, K. Barry, Brown JAL, M. J. Kerin

**Affiliations:** 1 Discipline of Surgery, Lambe Institute for Translational Research, School of Medicine, National University of Ireland Galway, Galway, Ireland; 2 Discipline of Surgery, Galway University Hospital, Galway, Ireland

**Keywords:** Breast cancer, Subtype, Discordance, Post-recurrence survival, Triple negative

## Abstract

**Background:**

Recent studies have shown that breast cancer subtype can change from the primary tumour to the recurrence. Discordance between primary and recurrent breast cancer has implications for further treatment and ultimately prognosis. The aim of the study was to determine the rate of change between primary and recurrence of breast cancer and to assess the impact of these changes on survival and potential treatment options.

**Methods:**

Patient demographics were collected on those who underwent surgery for breast cancer between 2001 and 2014 and had a recurrence with biopsy results and pathology scoring of both the primary and recurrence.

**Results:**

One hundred thirty two consecutive patients were included. There were 31 (23.5%) changes in subtype. Discordance occurred most frequently in luminal A breast cancer (*n* = 20), followed by triple negative (*n* = 4), luminal B (*n* = 3) and HER2 (n = 3). Patients who changed from luminal A to triple negative (*n* = 18) had a significantly worse post-recurrence survival (*p* < 0.05) with overall survival approaching significance (*p* = 0.064) compared to concordant luminal A cases (*n* = 46). Overall receptor discordance rates were: estrogen receptor 20.4% (*n* = 27), progesterone receptor 37.7% (*n* = 50) and HER2 3% (n = 4). Loss of estrogen receptor and progesterone receptor was more common than gain (21 vs. 6 (*p* = 0.04) and 44 vs. 6 (*p* = 0.01) respectively). Nine patients (6.8%) gained receptor status potentially impacting treatment options.

**Conclusion:**

Discordance in subtype and receptor status occurs between primary and recurrent breast cancer, ultimately affecting survival and potentially impacting treatment options.

**Electronic supplementary material:**

The online version of this article (10.1186/s12885-018-4101-7) contains supplementary material, which is available to authorized users.

## Background

Breast cancer is the second most common cancer worldwide and the most common cancer among women with an estimated 1.67 million women diagnosed annually, and the fifth leading cause of death from cancer overall [1]. Risk of recurrence and outcome in breast cancer have conventionally been stratified according to the tumour size, grade, nodal status and especially tumour subtype [2]. Breast cancer is a heterogeneous disease with 3 established immunohistochemical biomarkers: Estrogen Receptor (ER), progesterone receptor (PR) and HER2 (human epidermal growth factor 2) receptor. The presence or absence of these receptors defines the four distinct molecular subtypes of breast cancer- luminal A (ER/PR positive, HER2 negative), luminal B (ER and/or PR positive, HER2 positive), HER2 over-expressing (HER2 positive alone) and triple negative (negative for all 3 receptors) [3]. Each subtype exhibits distinct prognoses, rates of recurrence and different treatment strategies [4]. Following treatment, breast cancer recurrence can be classed as either loco-regional (LRR; confined to the ipsilateral breast/lymph nodes) or distant. Recurrence rates are influenced by the original breast cancer subtype, the specific therapy received and the response to the therapy [5]. Traditionally, recurrent tumours have been have been assumed to be biologically similar (the same subtype) to the primary tumour. Recent studies have demonstrated that hormonal and HER2 receptor status can change status between primary and recurrent breast cancer [6]. This can impact prognosis with loss of receptor status associated with a poorer prognosis [7, 8]. A change in receptor status could potentially lead to a change in treatment options, as patients whose recurrent tumour becomes hormone positive could be candidates for hormonal therapy and similarly patients who become HER2 positive may benefit from receiving Trastuzumab [9, 10].

The aim of our study was to identify subtype change in recurrent breast cancer at our institution, to assess the impact of discordance on patient outcomes, and to identify any potential changes in treatment due to a subtype change and if in reality patients who changed subtype experienced a change in treatment strategy.

## Methods

### Case selection

Data was collected on patients who had a recurrence of breast cancer following surgery +/− chemotherapy/hormonal therapy/radiotherapy at the Galway Hospitals group between 2001 and 2014. Loco-regional recurrence after surgery was defined as the appearance of tumour in the ipsilateral chest wall or axillary, internal mammary or supraclavicular lymph nodes while distant recurrence was defined as recurrence to distant organs, confirmed by pathologists report. Only patients who had clinical pathology scoring of receptor status of both the primary and recurrent cancer were included. Exclusion criteria included presentation with bilateral tumours, biopsy results that were incomplete, and pathologist report of the recurrence as a new primary tumour. PAS software was used to access pathology records with MOSAIQ software used to determine patient pathways and treatment.

### Pathology

Analysis of all samples was performed at the Pathology Laboratory, University Hospital Galway independently by clinical pathologists. Samples were obtained following surgery and at recurrence, with sufficient slides taken to perform all necessary immunohistochemical and pathological analysis. Samples were reviewed by a minimum of two pathologists, with an initial assessment from at least one primary reporting pathologist and a subsequent review performed by a pathologist at a multi-disciplinary meeting. The ER and PR receptor status were determined independently by clinical pathologists using immunohistochemistry [11] as per ASCO guidelines (ALLRED score > 2 or more than 1% stain positive). The HER2 receptor status was identified by Herceptest [12] as part of the routine clinical evaluation, with a score of 3+ considered positive. Any + 2 inconclusive results were confirmed using FISH testing [13] as per ASCO guidelines, with a HER2/CEP17 ratio greater than two considered amplified.

### Statistical analysis

Data analysis was performed using SPSS Version 21 (SPSS Inc., Chicago, IL). Overall survival and post-recurrence survival were estimated using the Kaplan-Meier product limit method. The log rank was used to determine any statistically significant differences in survival between the indicated groups. Comparative analyses were performed between groups using Chi-squared and T-tests. Statistical significance was accepted for *p* < 0.05.

### Ethics, consent and permissions

This study was conducted in accordance with the granted National University of Ireland Galway and University College Hospital Galway ethical approval. All patients had histologically confirmed breast cancer and all relevant clinic-pathological and demographic data were obtained from a prospectively maintained breast cancer database. This study used retrospectively collected, de-identified data, and no patients were involved.

## Results

### Patient demographics

One hundred thirty two patients were included. Mean age at diagnosis was 53.3 year (range 21–84). 58 patients (44%) had a loco-regional recurrence while 74 (56%) had a distant recurrence (Table 1). Bone was the most common distant recurrence (*n* = 27), followed by liver (*n* = 22) and lung (*n* = 16) (Table 2). 49 patients (37.2%) had breast-conserving surgery while 83 (62.8%) underwent mastectomy. 58 patients (44%) received neo-adjuvant chemotherapy prior to their primary surgery, with a mean time of 181 days (SD ±89.7) between diagnosis and surgery in this group. Mean time from diagnosis of primary disease to diagnosis of recurrence was 38.7 months (range 2–144 months) (Table 1). Mean overall survival (OS) was 60.1 months (SD ±38.2 months) while mean post-recurrence survival (PRS) was 20.8 months (SD ±21.1 months). The majority of patients in our cohort were stage 2 or stage 3 (41.6% and 29.5% respectively), grade 2 or 3 (40.1% and 52.3% respectively (Table 3).

**Table 1:** Cohort description

Patient Details	Total (*n* = 132)
Age at diagnosis: mean years (SD ±)	53.3 (SD ±13.6)
Time to recurrence: mean months (SD ±)	38.7 (SD ±27.7)
Recurrence location	
Loco-regional	58 (44%)
Distal	74 (56%)
Neoadjuvant Chemo Rx	
Received	58 (44%)
Did not receive	74 (56%)
Surgery	
Mastectomy	83 (62.8%)
Wide local excision	49 (37.2%)
Survival: Months	
Overall: mean (SD ±)	60 (38.3)
Post-recurrence survival: mean (SD ±)	20.7 (21.1)
Original subtype	
Luminal A	67 (50.7%)
Luminal B	10 (7.5%)
HER2	15 (11.3%)
Triple negative	40 (30.5%)
Recurrence subtype	
Luminal A	54 (40.9%)
Luminal B	9 (6.9%)
HER2	16 (12.1%)
Triple negative	53 (40.1%)

**Table 2:** Distant recurrence location & change in subtype

Distant recurrences (*n* = 74)	N (%)	Proportion that changed subtype
Bone	27 (36%)	4 (14%)
Liver	22 (30%)	5 (23%)
Lung	16 (22%)	4 (25%)
Lymph node distant	6 (8%)	1 (17%)
Brain	2 (3%)	0
Adrenal	1 (1.5%)	0

**Table 3:** Primary tumour features

Tumor details	n	(%)
Stage		
I	15	11.3%
II	55	41.6%
III A/B	39	29.5%
III C	23	17.4%
Grade		
1	10	7.6%
2	53	40.1%
3	69	52.3%
T		
1	37	28%
2	58	43%
3	34	25.7%
4	3	2.3%
N		
0	34	25.8%
1	46	34.8%
2	29	21%
3	23	14.4%

### Receptor discordance & survival

Rates of single receptor discordance for ER, PR and HER2 receptors were 20.4% (*n* = 27), 37.8% (*n* = 50), and 3% (*n* = 4) respectively (Table 4).Overall survival (OS) was comparable between the ER discordant group (*n* = 27) and the ER concordant group (*n* = 105), (60.2 vs. 59.3 months), while post-recurrence survival (PRS) was shorter in the discordant group, but this was not statistically significant (21.6 vs. 17.4 months, *p* = 0.36). There was no statistically significant difference in OS or PRS between the PR discordant (n = 50) and concordant (*n* = 82) groups (OS 67.1 vs. 55.7 months, *p* = 0.096, PRS 23.3 vs. 19.1 months, *p* = 0.096). In terms of HER2 receptor, there was a significant difference between the discordant (n = 4) and concordant (*n* = 128) groups in OS and PRS (OS 157 vs. 57 months, *p* < 0.05; PRS 60.7 vs. 19.5 months, *p* < 0.05). However, the very low numbers in the discordant group limit the value of this result. There was a statistically significant loss compared to gain of both ER and PR receptor status (ER loss *n* = 21 (15.9%) vs. gain *n* = 6 (4.5%), *p* = 0.04; PR *n* = 44 (33.2%) vs. n = 6 (4.5%), *p* = 0.01). Of the four HER2 receptor discordant cases, two gained and two lost receptor status, however these numbers are too low to draw statistical significance.

**Table 4:** Receptor discordance

ER	
Concordant	105 (79.6%)
Discordant	27 (20.4%)
Gain	6 (4.5%)
Loss	21 (15.9%)
PR	
Concordant	82 (62.1%)
Discordant	50 (37.8%)
Gain	6 (4.5%)
Loss	44 (33.2%)
HER2	
Concordant	128 (97%)
Discordant	4 (3%)
Gain	2 (1.5%)
Loss	2 (1.5%)
Subtype	N (%)
Concordant	101 (76.5%)
Discordant	31 (23.5%)

### Subtype discordance & survival

**Fig. 1:**
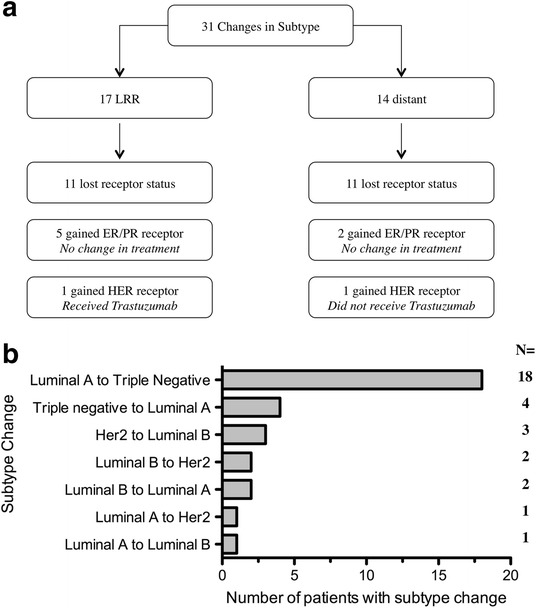
Cohort description. **a**. Total number of discordant cases and impact on treatment changes. **b.** Specific changes in subtype from primary to recurrence

**Fig. 2:**
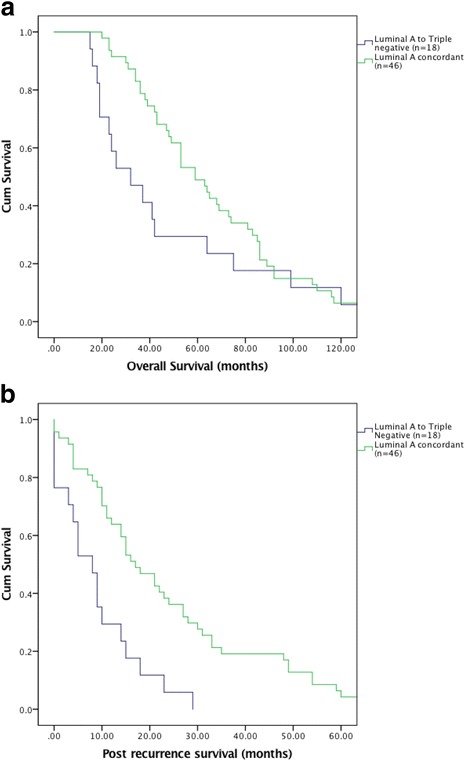
Luminal (**a**) to triple negative (*n* = 18) vs. triple negative concordant (*n* = 46)**.** A 10 year overall survival (*p* = 0.064). **b** 5 year post-recurrence survival (*p* < 0.05)

There were 31 patients (23.5%) who had a different subtype on recurrence, 17 were loco-regional recurrences and 14 were distant (Fig. 1). The group who changed subtype (*n* = 31) had a longer mean time to recurrence compared to the concordant group (*n* = 101) (44.9 vs. 36.9 months, *p* = 0.16) (Table 5). Recurrence location, type of surgery received and neo-adjuvant therapy were not associated with subtype change (*p* = 0.3, *p* = 0.83, *p* = 0.674 respectively) (Additional file 1: Table S1). A change from luminal A to triple negative (*n* = 18) subtype resulted in poorer 10 year OS versus the concordant luminal A group (*n* = 46) which approached statistical significance (46.8 vs. 67 months, *p* = 0.064) (Fig. 2A). Importantly, there was a statistically significant shorter 5 year PRS between the two groups, (8.6 vs. 22.5 months, *p* < 0.05) (Fig. 2B). Comparing patients who changed from triple-negative to luminal A (*n* = 4) to the concordant triple negative group (*n* = 35), there was no significant difference in 10 year OS (35 vs. 49 months, *p* = 0.378) or 5 year PRS (13.5 months vs. 14.2 months, *p* = 0.919) (Additional file 2: Figure S1).

**Table 5:** Impact of subtype change and gain in receptor status on survival

Patient Details	Total	Change subtype	Gain of Receptor
	(n = 132)	(n = 31) 23.5%	(*n* = 9) 6.8%
Survival: Months	N (%)	N (%)	N (%)
Overall: mean (SD ±)	60 (38.3)	64.9 (40.3)	76.9 (56.3)
Post-recurrence survival: mean (SD ±)	20.7 (21.1)	18.5 (22.8)	30.6 (30.3)

### Potential changes to treatment

In terms of changes in subtype that could potentially lead to a change in treatment, nine patients (6.8%) gained receptor status on recurrence. Seven patients went from HR negative to positive, with 6 patients going from ER negative to positive (ALLRED score 0 in the primary to > 2 in the recurrence). One patient went from PR negative to positive. Of these seven patients, five had a loco-regional recurrence and two had distant recurrences (one liver, one lung). None of these patients received additional endocrine therapy following the biopsy results of the recurrence. All nine patients are deceased with a mean OS of 52 months and a mean PRS of 21 months.

Two patients gained HER2 receptor status, both going from HER2 score of 0 on Herceptest of the primary to 1 of the recurrence, with both subsequently testing positive on FISH. One patient had a distant recurrence in bone, and was enrolled in the TRIO 022 trial [14], subsequently receiving Letrozole, Denosumab and a CDK inhibitor without receiving Trastuzumab. This patient is alive with an OS of 145 months and a PRS of 33 months. The other patient had a loco-regional recurrence and subsequently received 1 year of Trastuzumab. This patient is alive with an OS of 179 months and a PRS of 96 months.

In summary, only one patient in our study of nine who gained receptor status ultimately received additional targeted therapy.

## Discussion

In our single-centre analysis the rate of subtype change of was 23.5%, supporting previously published figs. [15, 16]. In terms of the specific changes in subtype, the most frequent change was from luminal A to triple negative, and this group had a significantly poorer 5 year PRS. Despite initially diverging OS, ultimately both groups have similar 10 year OS. Other studies have demonstrated a similar reduced survival in patients who change form HR positive to negative on recurrence [16–20].

Single receptor discordance was 20.4%, 37.8%, and 3% for ER, PR and HER2 receptor respectively, similar to that reported in a recent meta-analysis examining 48 papers, which reported pooled discordance rates of 20%, 33% and 8% for ER, PR and HER2 receptor [6]. HER2 receptor exhibits the lowest rate of discordance between primary and recurrence [21]. Loss of single receptor status was more common than gain for ER (*p* = 0.04) and PR (*p* = 0.01), in line with published data [22].

There are a number of possible aetiologies for receptor discordance. Firstly, variability exists in the reproducibility and accuracy of immunohistochemical staining [23]. There is also variability in sampling methods, for example fine needle aspiration or core biopsy versus surgical extraction in the primary tumour and in sampling of the recurrence that can contribute to the discrepancy. With the advent of next generation sequencing technology, it has become apparent that breast cancer demonstrates both intra-tumour and inter-tumour heterogeneity to a greater extent than previously understood. The discordance in receptor status may demonstrate clonal genome evolution [6, 24, 25] and the clone with the more aggressive phenotype could potentially initiate the micro-metastatic process [26]. Biological drift is another potential cause, for example selective eradication of ER/PR positive cells by hormonal therapy could leave behind a population of ER/PR negative cells that in time could metastasize [27]. Genuine switches in biology of the cancer appear to be a rare event based on currently available gene expression data [28, 29], however this does not exclude the potential for smaller scale genomic alterations and mutations [30]. Heterogeneity between patient’s primary and recurrence may be due to newly acquired biological characteristics that allow tumour cells to travel via the circulatory/lymphatic systems and to metastasize to new sites [31]. Change in receptor status may contribute to this increased capacity for invasion as endocrine and growth factor signalling pathways are implicated in invasion and metastasis [32, 33].

In terms of potential alterations to treatment and survival benefits of performing a recurrence biopsy, there is conflicting data with much of the literature being retrospective and examining small populations with variability in assay used, site of metastasis and definition of recurrence [7, 18, 34, 35]. Two prospective studies aimed to address these limitations - the BRITS study [36] in the United Kingdom which was carried out at 20 secondary care sites, and the DESTINY study [10] conducted at a single centre in Toronto, Canada. Both were conducted using similar eligibility and exclusion criteria. A pooled analysis of the two studies examined the proportion of patients who underwent a change in management based on the results of the recurrence biopsy [37]. 289 patients underwent biopsy of recurrence, consisting of 48% loco-regional recurrences and 52% distal metastases. 14.2% of patients had a change in management based on their results. However, on further analysis, half of the changes in treatment regime were due to loss of receptor status, new primary diagnosis or benign disease on biopsy. In total only 7.1% of patients had a treatment added due to gain in receptor status.

In terms of the effect that changing management had on patient outcomes, the results were unclear and only the DESTINY trial looked at overall survival. There was no significant association between overall survival and discordance (median OS 27.6 vs. 30.2 months in the concordant and discordant groups respectively). Other retrospective studies have identified a change in management plan in 12–20% of patients where there was a gain in receptor status [15, 35, 38].

Current guidelines by the American Society of Clinical Oncology (ASCO) [39] advise offering biopsy where feasible to patients with recurrence for receptor status. Treatment should be guided preferentially by the ER/PR/HER2 status of the recurrence if justified by the clinical scenario and conforming to the patient’s wishes. The panel’s recommendations are deemed to be “moderate” due to the paucity of clinical evidence demonstrating that altering therapy based on receptor change has significant health outcomes. A number of barriers exist to routine biopsy of tumour recurrence – it may not be technically feasible or safe to perform, there is a 2% risk of major complications [40], and the patient or physician may decide against it.

Limitations of our study include the relatively small sample size. The retrospective nature of the study made it difficult to accurately collate data on patient’s precise treatment regimes. Furthermore, as discussed above technical misclassification is a significant contributor to receptor discordance. Gain in receptor status may be attributable to this misclassification as opposed to a genuine change in tumour biology [41, 42]. It may be beneficial to carry out an independent re-review of the pathology slides from this study to identify what proportion of subtype change was due to this misclassification.

## Conclusions

In summary, our study demonstrates the discordance of receptor and subtype between primary and recurrent breast cancer at our institution. It highlights the importance of performing a biopsy of recurrent breast cancer, due to the implications that change in subtype has on survival. Further research is required to investigate the aetiology and biology of subtype discordance and the optimal strategy for treatment change based on this discordance. Our results highlight the need for a prospective, multicentre trial collecting data on patients who experience recurrence (including routine biopsy’s of recurrence), to establish if all recurrent patients should be biopsied, or only a subset of patients most likely to benefit from additional treatment options.

## Additional files

Additional file 1: **Table S1.** Impact of Location/Neoadjuvant chemotherapy/Surgery on discordance. Quantifying: Recurrence location, Neoadjuvant Chemo Rx, Surgery, Change subtype, Gain of Receptor. (DOCX 24 kb)

Additional file 2: **Figure S1.** Triple negative to Luminal A (*n* = 4) vs. triple negative concordant (*n* = 35). A 10 year overall survival (*p* = 0.378). B 5 year post recurrence survival (*p* = 0.919). (TIFF 542 kb)

## Abbreviations

CDK: Cyclin dependent kinase;
ER: Estrogen receptor
FISH: Fluorescence in situ hybridization
HER2: Her2 over-expressing breast cancer
Her2: Human epidermal growth factor receptor 2
HR: Hormone receptor
HR: Hormone receptor
IHC: Immunhistochemical
OS: Overall survival
PR: Progesterone receptor
PRS: Post-recurrence survival
TN: Triple Negative
