# Correction to: Breast cancer subtype discordance: impact on post-recurrence survival and potential treatment options

**DOI:** 10.1186/s12885-018-4174-3

**Published:** 2018-03-13

**Authors:** Peter F. McAnena, Andrew McGuire, A. Ramli, C. Curran, C. Malone, R. McLaughlin, K. Barry, James A. L. Brown, M. J. Kerin

**Affiliations:** 1 Discipline of Surgery, Lambe Institute for Translational Research, School of Medicine, National University of Ireland Galway, Galway, Ireland; 2 Discipline of Surgery, Galway University Hospital, Galway, Ireland

## Correction to: BMC cancer (2018) 18:203 DOI: 10.1186/s12885-018-4101-7

It has been highlighted that the original manuscript [1] contains a typesetting error regarding the authorship and did not mention the name of Andrew McGuire. The authorship was incorrectly captured as Peter F. McAnena^1^, James AL Brown^1^, A. Ramli^1^, C. Curran^1^, C. Malone^2^, R. McLaughlin^2^, K. Barry^2^, Brown JAL^1^* and M. J. Kerin^1^ in the original article. The correct authorship is: Peter F. McAnena^1^, Andrew McGuire^1^, A. Ramli^1^, C. Curran^1^, C. Malone^2^, R. McLaughlin^2^, K. Barry^2^, James A.L. Brown^1^* and M. J. Kerin^1^ . The original article has been updated.
