# Supplementary material for: Breast cancer subtype discordance: impact on post-recurrence survival and potential treatment options
Source: BMC Cancer. 2018 Feb 20;18:203. doi: 10.1186/s12885-018-4101-7 (PMC5819681; doi:10.1186/s12885-018-4101-7)
Supplement: Supplementary file 1 — Table S1. Impact of Location/Neoadjuvant chemotherapy/Surgery on discordance. Quantifying: Recurrence location, Neoadjuvant Chemo Rx, Surgery, Change subtype, Gain of Receptor. (DOCX 24 kb) [file 12885_2018_4101_MOESM1_ESM.docx]

**Table Supplemental 1 – Impact of Location/Neoadjuvant chemotherapy/Surgery on discordance**

| **Patient Details** | **Total**  **(n=132)** | **Change subtype**  **(n=31) 23.5%** | **Gain of Receptor**  **(ER, PR or HER2)**  **(n=9) 6.8%** |
| --- | --- | --- | --- |
| **Recurrence location** | **N (%)** | **N (%)** | **N (%)** |
| Loco-regional  Distal  **P value** | 58 (44%)  74 (56%) | 17 (29.3%)  14 (18.9%)  **0.299** | 6 (10.5%)  3 (4%)  **0.174** |
| **Neoadjuvant Chemo Rx** | **N (%)** | **N (%)** | **N (%)** |
| Received  Did not receive  **P value** | 58 (44%)  74 (56%) | 14 (24.1%)  17 (23%)  **0.674** | 1 (1.7%)  8 (11.1%)  **0.077** |
| **Surgery** | **N (%)** | **N (%)** | **N (%)** |
| Mastectomy  Wide local excision  **P value** | 83 (62.8%)  49 (37.2%) | 21 (25.3%)  10 (20.4%)  **0.83** | 7 (8.4%)  2 (4.1%)  **0.48** |
