# Supplementary figures and images for: Breast cancer subtype discordance: impact on post-recurrence survival and potential treatment options
Source: BMC Cancer. 2018 Feb 20;18:203. doi: 10.1186/s12885-018-4101-7 (PMC5819681; doi:10.1186/s12885-018-4101-7)

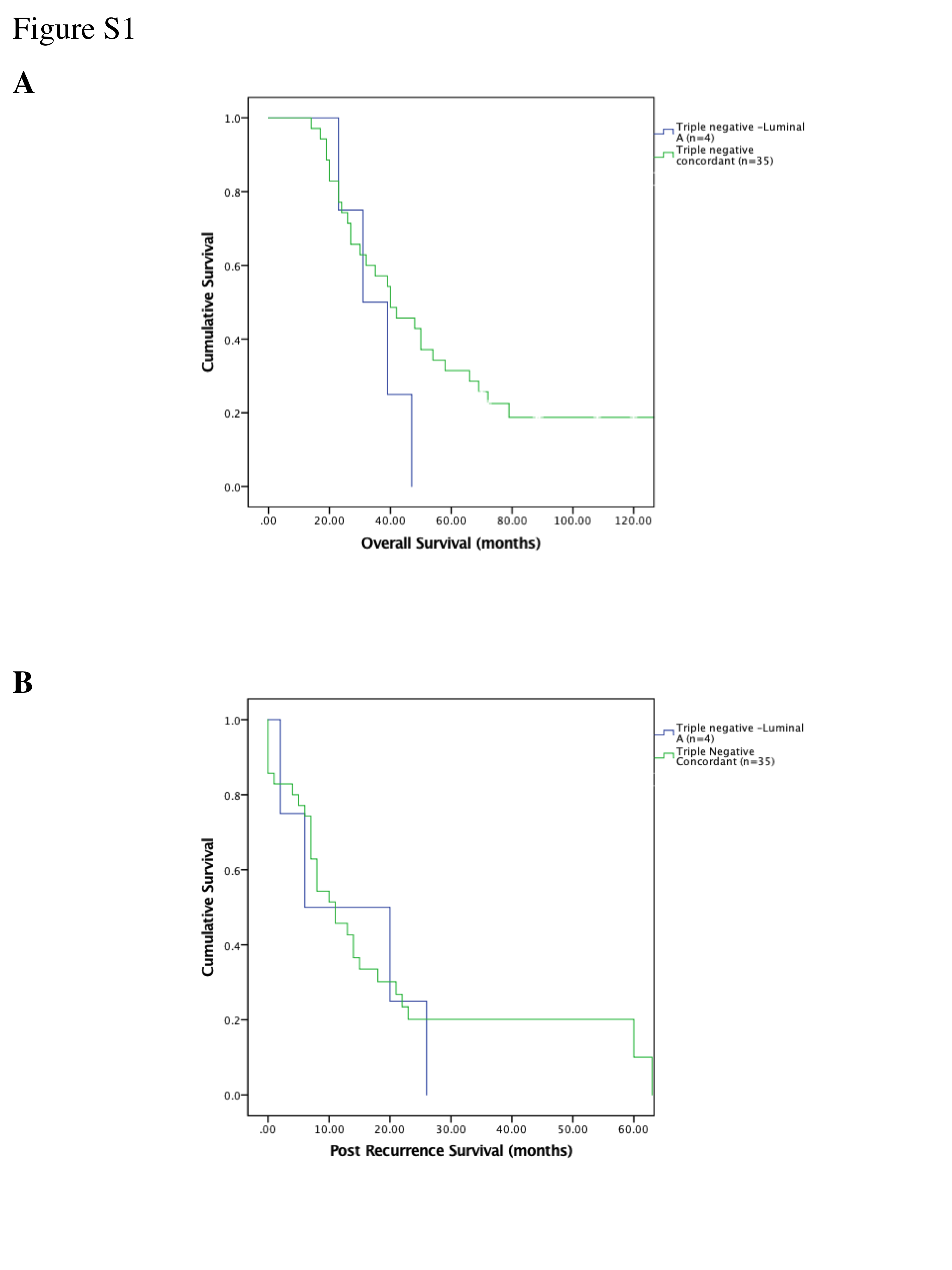

Supplement: Supplementary file 2 — Figure S1. Triple negative to Luminal A (n = 4) vs. triple negative concordant (n = 35). A 10 year overall survival (p = 0.378). B 5 year post recurrence survival (p = 0.919). (TIFF 542 kb) [file 12885_2018_4101_MOESM2_ESM.tif]
